# Engaging Ly‐6A/Sca‐1 triggers lipid raft‐dependent and ‐independent responses in CD4^+^ T‐cell lines

**DOI:** 10.1002/iid3.182

**Published:** 2017-06-28

**Authors:** Melissa A. Lang, Sultan A. Jenkins, Phillip Balzano, Adeyinka Owoyele, Akshay Patel, Anil K. Bamezai

**Affiliations:** ^1^ Department of Biology Villanova University Villanova Pennsylvania

**Keywords:** Apoptosis, CD4^+^ T cells, cell cycle, GPI‐anchored, lipid raft, Ly‐6A, membrane order, Sca‐1

## Abstract

**Introduction:**

The lymphocyte antigen 6 (Ly‐6) supergene family encodes proteins of 12–14 kda in molecular mass that are either secreted or anchored to the plasma membrane through a glycosyl‐phosphatidylinisotol (GPI) lipid anchor at the carboxy‐terminus. The lipidated GPI‐anchor allows localization of Ly‐6 proteins to the 10–100 nm cholesterol‐rich nano‐domains on the membrane, also known as lipid rafts. Ly‐6A/Sca‐1, a member of Ly‐6 gene family is known to transduce signals despite the absence of transmembrane and cytoplasmic domains. It is hypothesized that the localization of Ly‐6A/Sca‐1 with in lipid rafts allows this protein to transduce signals to the cell interior.

**Methods and Results:**

In this study, we found that cross‐linking mouse Ly‐6A/Sca‐1 protein with a monoclonal antibody results in functionally distinct responses that occur simultaneously. Ly‐6A/Sca‐1 triggered a cell stimulatory response as gauged by cytokine production with a concurrent inhibitory response as indicated by growth inhibition and apoptosis. While production of interleukin 2 (IL‐2) cytokine by CD4^+^ T cell line in response to cross‐linking Ly‐6A/Sca‐1 was dependent on the integrity of lipid rafts, the observed cell death occurred independently of it. Growth inhibited CD4^+^ T cells showed up‐regulated expression of the inhibitory cell cycle protein p27^kip^ but not of p53. In addition, Ly‐6A/Sca‐1 induced translocation of cytochrome C to the cytoplasm along with activated caspase 3 and caspase 9, thereby suggesting an intrinsic apoptotic cell death mechanism.

**Conclusions:**

We conclude that opposing responses with differential dependence on the integrity of lipid rafts are triggered by engaging Ly‐6A/Sca‐1 protein on the membrane of transformed CD4^+^ T cells.

## Introduction

Mouse Ly‐6A, also known as Sca‐1 (Stem cell antigen 1), is a GPI‐anchored membrane protein and the prototypic member of the lymphocyte antigen 6 (Ly‐6) supergene family. These proteins were first described as allo‐antigens expressed on activated murine lymphocytes 1. The Ly‐6 proteins are expressed in a wide range of organisms ranging from *C. elegans* to humans, and across tissue types as variable as stem cells, lymphocytes, neurons, and muscle cells 2, 3. A number of Ly‐6 proteins, including Ly‐6A/Sca‐1, have cell‐cell adhesion properties in a variety of cell types 4, 5, 6, 7, 8. Cross‐linking of Ly‐6 proteins with anti‐Ly‐6 monoclonal antibodies alone is sufficient to induce cell activation in transformed T cells 9, 10, but additional co‐stimulation is required to activate primary mouse CD4^+^ T lymphocytes 9, 10. Expression of Ly‐6A/Sca‐1 regulates signaling through the antigen receptor on CD4^+^ T cells and their cytokine responses 11, 12, 13. The Ly‐6 gene locus also influences susceptibility to mouse adeno virus in murine models, West Nile virus, HIV‐1, and several other DNA and RNA viruses 14, 15, 16, 17. While various members of Ly‐6 family are recognized for their role in cytokine responses by T cells, the full spectrum of responses, and the contribution of lipid rafts to signaling initiated by engaging Ly‐6A/Sca‐1 is unknown.

Ly‐6A/Sca‐1 signals to the cell interior despite the absence of a transmembrane and cytoplasmic tail. Inclusion of the lipid anchored Ly‐6A/Sca‐1 protein in the lipid rafts on the plasma membrane raises the possibility that this tail‐less protein may possibly co‐opt these signaling platforms to transduce signals. Lipid rafts are dynamic nano‐domains on the plasma membrane that play an essential role in signal transduction by providing a platform to assemble signaling receptors, enzymes, and adaptor proteins 18. We report here that engaging Ly‐6A/Sca‐1 protein on transformed murine T cells signals for cytokine response, growth inhibition, and apoptosis. While the interleukin 2 (IL‐2) cytokine response is dependent on the integrity of the lipid rafts, the apoptotic cell death triggered by Ly‐6A/Sca‐1 is lipid raft independent. High expression of Ly‐6A/Sca‐1 observed on transformed cells, and its growth inhibition and apoptosis triggered in immortalized T cell lines by engaging this protein, suggests its promise as a potential tumor antigen target.

## Materials and Methods

### Cell culture

YH16.33, MVB2, KQ23.37.7 and D10.G4, T‐T hybridomas, (generous gift from Ken Rock) 19 were cultured in RPMI 1640‐GlutaMAX™ (Invitrogen, Carlsbad, CA) supplemented 0.01 M HEPES, Antibiotics/Antimycotics (Invitrogen), Non‐Essential Amino Acids (Irvine Scientific, Santa Ana, CA), 0.25 mM β‐mercaptoethanol (Sigma–Aldhrich, St. Louis, MO), and 10% FBS (Atlanta Biologics, Atlanta, GA). The cell lines were incubated at 5% CO_2_ and 37°C under humidified conditions.

### Cell treatments

YH16.33, KQ23.37.7, and D10.G4 cells were incubated with either anti‐Ly‐6A (8G12) 20 or anti‐CD3ϵ (145‐2C11) 21 for 4–48 h at 37°C in humidified 5% CO2 incubator. In some treatments Nutlin‐3a (Sigma–Aldrich, St. Louis, MO), at 10 µg/ml (17.2 µM) final concentration was added to the cultures. 293T cell line (American Type Culture Collection [ATCC] Manassas, VA) was also cultured in this manner to act as a positive control for p53.

7 keto‐cholesterol (7‐KC) and MβCD complexes were generated and incorporated into the plasma membrane by following a previously published protocol 22. Briefly, cells were treated with a mixture of an appropriate concentration of 7‐KC (Sigma–Aldrich, St‐Louis, MO) ranging from 58 to 14 μM and a fixed concentration (0.3 mM) of MβCD (Sigma–Aldrich). 7‐KC‐MβCD complexes were added to YH16.33 cells for 15 min at 37°C in 5% CO_2_ incubator. Cells were washed to remove 7‐KC complexes by centrifugation and re‐suspended in culture media and examined for responses through Ly‐6A and anti‐CD3ϵ. Low concentration (0.3 mM) of MβCD does not disrupt lipid rafts 22.

### Cellular proliferation—MTS assay

Cell proliferation was measured using the CellTiter 96® Aqueous One Solution Cell Proliferation Assay (Promega Corp., Madison, WI) as per the manufacturer's instructions. Briefly, YH16.33 (5 × 10^3^ per well) cells were seeded in a 96‐well plate with 100 μl of fresh RPMI 1640‐GlutaxMAX™ cell culture media. The cells were either cultured for 4, 8, 24, or 48 h in media alone that served as negative control or media containing Ly‐6A/Sca‐1 monoclonal antibody at 4 μg/ml concentration. A total of 20 µl of CellTiter 96® AQueous One Solution Reagent (Promega Corp., Madison, WI) was added to each well, and then the plate was incubated at 37°C in humidified 5% CO_2_ incubator for 1 h. The absorbance was read at 490 nm using a 96‐well plate reader.

### Cytokine assays

To quantify IL‐2 in anti‐Ly‐6A and anti‐CD3ϵ treated YH16.33 cells, the top 100 μl of supernatants was harvested at both 24 h time points and then frozen at −20°C for ELISA analysis. IL‐2 assay kit was used (BD Bioscience, San Jose, CA). Briefly, 24 h before the cytokine assay was carried out, ELISA plate (Costar, USA) was coated with 50 μl/well capture antibody diluted in 1:250 in coating buffer (carbonate/bicarbonate, pH 9.0) and stored at 4°C overnight as per instructions from the vendor (BD Biosciences). After overnight incubation, the unbound capture antibody from the 96‐well plate was removed by dumping the contents, and each well was subsequently washed with 100 ul of Phosphate with 0.05% Tween 20 (Sigma–Aldrich, St. Louis, MO). This was then followed by a blocking step; 100 ul of blocking buffer (PBS without Tween‐20 +10% FCS) and incubated at room temperature for 30 min. The assay supernatants and standards were then added to the appropriate wells and incubated for 90 min at room temperature. After this incubation, the contents of the plate were dumped and washed five times with wash buffer. A total of 50 μl of detector reagent (biotinylated anti‐IL‐2 detection antibody at 1:1000 and Avidin HRP enzyme [BD Biosciences] at 1:250 dilution) for the cytokine being detected was added to the plate and incubated for 30 min at 4°C with the plate covered with aluminum foil. After 30 min, the detector was dumped and the wells were washed seven times with wash buffer with each wash done for about 30 s. Assay was developed with substrate and chromogen solution (KPL, Milford, MA) in a 1:1 ratio and 100 μl of the mixture was added to each well. To serve as a blank, two untreated wells contained substrate. The assay was developed in dark for about 30 min. The plate was then read at 405 nm using Spectramax 190 plate reader (Molecular Devices, Sunnyvale, CA). The data for each treatment was done in triplicate. In the final analysis of data, an average of the three values was taken and the graphs that were plotted shows the average values.

### Western blot analysis

YH16.33 cells were lysed in Radioimmunoprecipitation Assay (RIPA) Buffer (150 mM sodium chloride, 1.0% NP‐40, 0.5% sodium deoxycholate, 0.1% sodium dodecyl sulphate, 50 mM Tris, pH 8.0) (Sigma–Aldrich, St‐Louis, MO) with 1X Phosphatase and Protease Inhibitor Cocktail (Thermo Fischer Scientific, Waltham, MA) on ice for 15 min. Cell debris was removed by centrifugation at 16,000*g* for 10 min at 4°C, and the supernatant was immediately transferred to a fresh tube. The protein concentration of each sample was determined using Pierce BCA Protein Assay Kit (Thermo Fisher Scientific, Waltham, MA). Proteins (30 μg) were subjected to 4–15% SDS–PAGE at 200 V for 30 min and then transferred on to a PVDF (polyvinylidene difluoride) or nitrocellulose membrane at 100 V for 1 h. After blocking with 5% skim milk in TBST (20 mM Tris, pH 7.5, 150 mM NaCl, 0.1% Tween‐20) for 1 h, the membrane was probed overnight at 4°C with the following primary monoclonal antibodies at a 1:1000 dilution: anti‐caspase‐3 (clone 8G10), ‐8 (clone D35G2), ‐9 (clone C9), anti‐β‐actin (clone 13E5), anti‐p27^kip^ (clone D37H1), anti‐p53 (clone 7F5) (Cell Signaling Technology Inc., Danvers, MA). Bound antibodies were detected using a horseradish peroxidase‐conjugated to either anti‐rabbit IgG or anti‐mouse IgG secondary antibody at a 1:1000 dilution (Cell Signaling Technology Inc., Danvers, MA) followed by detection using SuperSignal West Pico Chemiluminescent (Thermo Fisher Scientific, Waltham, MA) or ECL substrate (EMD Millipore Corp., Billerica, MA).

### Flow cytometry

#### Active caspase‐3 staining

The detection of active caspase‐3 was carried out using The CaspGLOW™ Fluorescein Active Caspase‐3 Staining Kit (BioVision, Mountain View, CA) per the manufacturer's instructions. Briefly, YH16.33 cells were incubated in media alone (Non‐treated negative control) or with 4 μg/ml Ly‐6A monoclonal antibody (mAb) for 4, 8, 24, or 48 h. Afterwards, 300 μl of the cell culture was incubated with 1 μl of fluorescein isothiocyanate (FITC)‐DEVD‐FMK (BioVision) marker for 1 h at 37°C in humidified 5% CO_2_ incubator. The cells were then centrifuged at 3000 rpm for 5 min, the supernatant removed, and the cells were re‐suspend in 0.5 ml of Wash Buffer. The cells were then centrifuged again, then resuspended in 300 ml of Wash Buffer, and then analyzed by FACSCalibur flow cytometer (BD Biosciences, Palo Alto, CA) using the FL‐1 channel.

### Flow cytometry and cytochrome C staining

The intracellular cytochrome staining was carried out according to a published study 23. Briefly, YH.33 cells were treated with 100 µl of a digitonin solution (50 µg/ml in PBS with 100 mM KCl) for 5 min on ice. The cells were then fixed with 4% paraformaldehyde solution (Electron Microscopic Sciences, Hatfield, PA) made in PBS for 20 min. Paraformaldehyde was washed three times, and treated with a blocking buffer consisting of 3% BSA, 0.05% saponin, and made in PBS for 1 h. The cells incubated with a FITC‐tagged monoclonal antibody against cytochrome C (clone 6H2.B4) (BioLegend, San Diego, CA), at 1:200 monoclonal dilution and were then analyzed using Flow Cytometry.

### Flow cytometry and staining with Di‐4‐ANEPPDHQ

Lymph node cells were stained with fluorescent membrane dye, di‐4‐ANEPPDHQ (Invitrogen–Life Technologies, Grand Island, NY) at 0.5 μM final concentration for 20 min at room temperature as previously reported 22. Labeled cells were analyzed by FACSCalibur flowcytometer (BD Biosciences, East Rutherford, NJ) using 488 nm excitation lasers. Emission from the labeled cells was recorded at wavelengths 570 nm (FL2 channel), 630 nm (FL3 channel). Folowing formula was used to assess generalized polarization (GP) values = I_570_−I_630_/I_570_ + I_630;_ I = mean fluorescence intensity.

### Statistical analyses

All Statistical analysis, except where indicated in the legend, was done using one‐way ANOVA testing. Either One‐Way ANOVA or non‐parametric Krusal–Wallis ANOVA was initially used to examine the overall variance, followed by either Tukey HSD or MCTP testing respectively in RStudio to determine where differences in the data lie. Results were considered significant if the *p*‐value was less than 0.05.

### Ethics statement

No animals or human subjects were used or involved in our study.

## Results

### Engaging Ly‐6A/Sca‐1 with anti‐Ly‐6A/Sca‐1 mAb promotes cytokine production in YH16.33 T cells dependent on the integrity of lipid raft‐based membrane order

**Figure 1:**
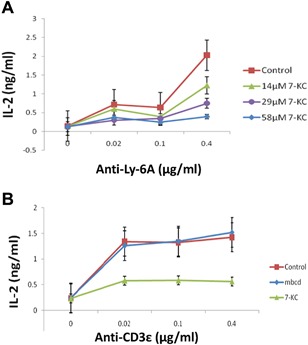
Effect of 7‐Keto Cholesterol on IL‐2 production by YH16.33 CD4^+^ T cell line in response to anti‐Ly‐6A/Sca‐1 and anti‐CD3ϵ antibodies. (A) Cells were treated with different concentrations of 7‐KC ranging from14 μM (green), 29 μM (purple), 58 μM (blue) for 30 min at 37°C to disrupt lipid raft‐based membrane order followed by stimulation with anti‐Ly6A (8G12) mAb. The antibody supernatant with concentration of antibodies equivalent to 0.4, 0.1, and 0.02 μg/ml was used. The level of IL‐2 produced was measured for each treatment. (B) Inhibition of IL‐2 response by 7‐KC treatment of YH16.33 CD4^+^ T cells in response to anti‐CD3ϵ (145‐2C11) mAb. The cells were treated with 0.5 mM mβcd (blue), 29 μM 7‐KC (green), and or left untreated (brown) for 30 min at 37°C followed by stimulation with anti‐CD3ϵ (145–2C11) antibody under experimental conditions described above (A) and materials and methods section. Each data point represents a mean of duplicate trials for each treatment. Data shown is representative of six experiments. Mean IL‐2 response for three different concentrations of antibody treatment from six independent sets of experiments is shown. Error bars are standard error, *p* values reported for each 7‐KC concentration in 0.4 μg/ml anti‐Ly‐6A/Sca‐1 antibody treatment was ascertained by comparing with no‐treatment controls. Paired T test analyses show: Control versus 14 µM 7‐KC: *t*(10) = 3.4556; *p* = 0.0062; standard error of difference = 0.131; Control versus 29 µM: *t*(10) = 4.9419; *p* = 0.0006; standard error of difference = 0.150; Control versus 58 µM: *t*(10) = 6.2821; *p* = 0.0001; standard error of difference = 0.145.

Engaging Ly‐6A/Sca‐1 with anti‐Ly‐6A/Sca‐1 mAbs is known to activate CD4^+^ T cell lines resulting in gene transcription and translation of multiple cytokines 9, 10, 19, 20. We examined the role of lipid raft‐based membrane order in CD4^+^ T cell response generated after engaging Ly‐6A/Sca‐1 protein with anti‐Ly‐6A mAB (8G12). Membrane order was disrupted by inserting an oxyseterol, 7‐Keto Cholesterol (7‐KC) in the membrane of a CD4^+^ T cell line as reported previously 22. Exposure to 7‐KC resulted in loss of lipid raft‐based membrane order in a concentration‐dependent manner, (Supplementary Fig. S1) similar to what was observed with primary mouse CD4^+^ T cells reported previously 22. YH16.33 T cells exposed to different concentrations of 7‐KC, prior to engaging Ly‐6A/Sca‐1 protein with a Ly‐6A/Sca‐1 specific monoclonal antibody (mAb) 8G12, produced significantly lower IL‐2 (Fig. 1A). The amount of IL‐2 produced with highest concentration of the anti‐Ly‐6A/Sca‐1 antibody was inversely proportional to the 7‐KC concentration with >80% inhibition observed with highest 58 μM concentration used (Fig. 1A). Similarly, in response to stimulation through the (T cell receptor) TCRαβ/CD3 complex, 7‐KC treated YH16.33 cells produced lower levels of IL‐2 than either the untreated or vehicle (mβCD) treated cells (Fig. 1B). 7‐KC treated cells expressed similar levels of Ly‐6A/Sca‐1, lymphocyte function antigen 1 (LFA‐1), TCRβ, and CD3ϵ proteins on the cell surface as the untreated controls (Supplementary Fig. S2) and therefore does not account for the observed altered IL‐2 responses through the antigen receptor complex and Ly‐6A/Sca‐1 protein.

### Anti‐Ly‐6A/Sca‐1 mAb induces apoptotic cell death in YH16.33 T cells independent of lipid raft‐based membrane order

**Figure 2:**
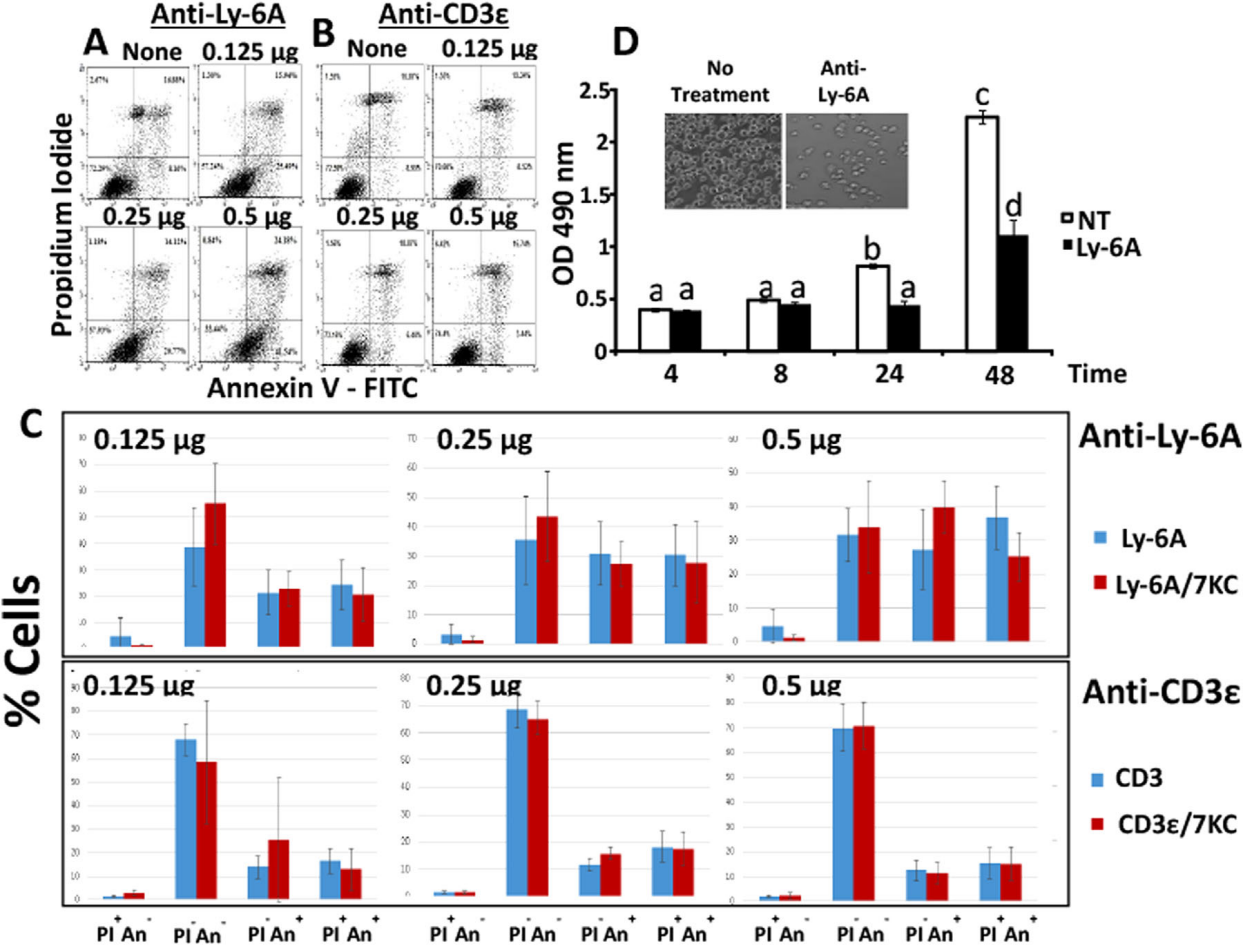
Quantification of apoptosis and growth inhibition induced by anti‐Ly‐6A/Sca‐1 antibody. YH16.33 cells treated with 7‐KC were incubated with either anti‐Ly‐6A/Sca‐1 (8G12) (Panel A) or anti‐CD3ϵ (145‐2C11) (Panel B) for 24 h and cell cultures were stained with annexin V‐FITC and propidium iodide (PI) followed by their flow cytometeric analyses. Live (PI^−^AnV^−^), apoptotic (PI^−^AnV^+^) and dead (PI^+^AnV^+^) cells without any gating were enumerated. Mean survivorship and cell death with three different concentrations of anti‐Ly‐6A/Sca‐1 (8G12) (Panel C—top) and control anti‐CD3ϵ (145‐2C11) (Panel C—bottom) antibody treatment from three independent sets of experiments is shown. Additionally, these experiments were also carried out in YH16.33 treated with 58 μM 7‐KC to disrupt their lipid raft‐based membrane order (Panel C—top and bottom). Error bars are standard error, *p* values ascertained by one way ANOVA analysis, no statistical significant differences between 7‐KC‐treated and untreated groups were observed (*p *> 0 .05). Ly‐6A/Sca‐1 mAb inhibits cell proliferation of YH16.33 cells as well (Panel D and inset panel). YH16.33 was cultured either with RPMI media alone (Non‐treated control group, NT) or with 4 μg/ml anti‐Ly‐6A/Sca‐1 antibody (8G12). Cells were harvested and analyzed by the MTS assay at the indicated time points. Absorbance at 490 nm was measured in triplicate samples. The data represent the mean ± S.D. of three independent experiments. Groups denoted with different letters are statistically different (*p* < 0.01). *p* values ascertained by one way ANOVA analysis Inset panel in D shows images of un‐treated (left) or anti‐Ly‐6A/Sca‐1 (8G12) (right) treated antibodies (8G12). The cells were imaged after culturing them with 8G12 or left untreated for 24 h (160X).

While the activated YH16.33 cells produced IL‐2, a signature cytokine for T cell response, visual examination of cells suggested cell death and growth inhibitory effects of the antibody which prompted further investigation. To determine and quantify the effect of cross linking Ly‐6A/Sca‐1 proteins on cell death and cellular proliferation, YH16.33 cells were treated with different amounts of anti‐Ly‐6A/Sca‐1 antibody at varying time periods before assessing their survival and cellular proliferation. Anti‐Ly‐6A/Sca‐1‐treated cells were assessed for apoptosis by staining with Annexin V‐FITC and PI. Figure 2A shows YH16.33 cells undergo apoptotic cell death in the presence of anti‐Ly‐6A/Sca‐1 antibody in a concentration‐dependent manner as examined by flow cytometer. All cells (without gating) were analyzed after staining with Annexin V‐FITC and PI. Untreated and anti‐Ly‐6A/Sca‐1 treated YH16.33 cell cultures showed 8% and 41% apoptotic (Annexin V^+^PI^−^) cells, respectively (Fig. 2A). In contrast, apoptotic cell death in the YH16.33 control cultures treated with anti‐CD3ϵ antibody did not deviate significantly from the untreated YH16.33 cells (less than 8.9%) (Fig. 2B). Viability of YH16.33 cells at the beginning of the cell culture ranged from 93% to 97%.

Ligand induced crosslinking of “death receptors,” such as Fas, results in their aggregation and formation of Death‐Inducing Signaling Complex (DISC) which in turn are recruited to lipid rafts 24, 25. Ly‐6A/Sca‐1 protein, because of its GPI‐anchor, is housed in cholesterol and saturated lipid‐rich lipid rafts as well on the plasma membrane 26, 27. Therefore, we tested the role of lipid rafts in Ly‐6A/Sca‐1‐mediated cell death. Cells were treated with 7‐ketocholesterol to disrupt the integrity of raft nano‐domains as per previously published reports 22, 28, 29 and then incubated with anti‐Ly6A mAb for 24 h. Such treatment caused an increase in apoptosis in anti‐Ly‐6A/Sca‐1 mAb concentration‐dependent manner but independent of the treatment with 7‐KC. As seen in Figure 2C, even the lowest concentration of the Ly‐6A/Sca‐1 mAb induced apoptosis in YH16.33 T cell line four times above the background despite 7‐KC treatment. An average of 31.6% (*n* = 3) cells were scored apoptotic at 0.5 μg of anti‐Ly‐6A/Sca‐1 antibody. To investigate the specificity of this response, we tested for the effects of cross‐linking the TCRαβ/CD3 complex with anti‐CD3ϵ mAb. As shown in Figure 2C, apoptotic cells ranged from 4% to 10% of the population over the same range of antibody concentration making it indistinguishable from the background. We did not observe statistically significant differences between 7‐KC‐treated and untreated groups (*p *> 0.05). Our data suggest that apoptosis induced by engaging Ly‐6A/Sca‐1 is specific, and occurs independent of the integrity of the lipid rafts. In contrast, as shown in Figure 1, IL‐2 response generated by YH16.33 under similar conditions is dependent on lipid raft integrity.

To quantify the visual growth inhibitory effects of engaging Ly‐6A/Sca‐1 on YH16.33 cells (inset in Fig. 2D), YH16.33 cells were treated with 8G12 mAb and cell cultures were incubated for 4–48 h before cellular proliferation was assessed by 3‐(4,5‐dimethylthiazol‐2‐yl)‐5‐(3‐carboxymethoxyphenyl)‐2‐(4‐sulfophenyl)‐2H‐tetrazolium) (MTS) assay with end point measurement of optical density of cell cultures at 490 nm. Figure 2D shows that Anti‐Ly‐6A/Sca‐1 mAb, 8G12, treatment inhibited the proliferation of YH16.33 cells in a time‐dependent manner. The growth inhibition of YH16.33 cells was more pronounced at 24 and 48 h time points (Fig. 2D). Engaging TCRαβ/CD3 complex with anti‐CD3ϵ, a stimulatory control antibody, resulted in IL‐2 production without any growth inhibitory affects (Figs. 1 and S3). Effects of anti‐Ly‐6A/Sca‐1 was specific since the growth inhibitory effect of anti‐Ly‐6A/Sca‐1 was not observed on MVB2 cells, a T cell line derived from YH16.33 lacking expression of Ly‐6A/Sca‐1 on the surface (Supplementary Fig. S3).

**Figure 3:**
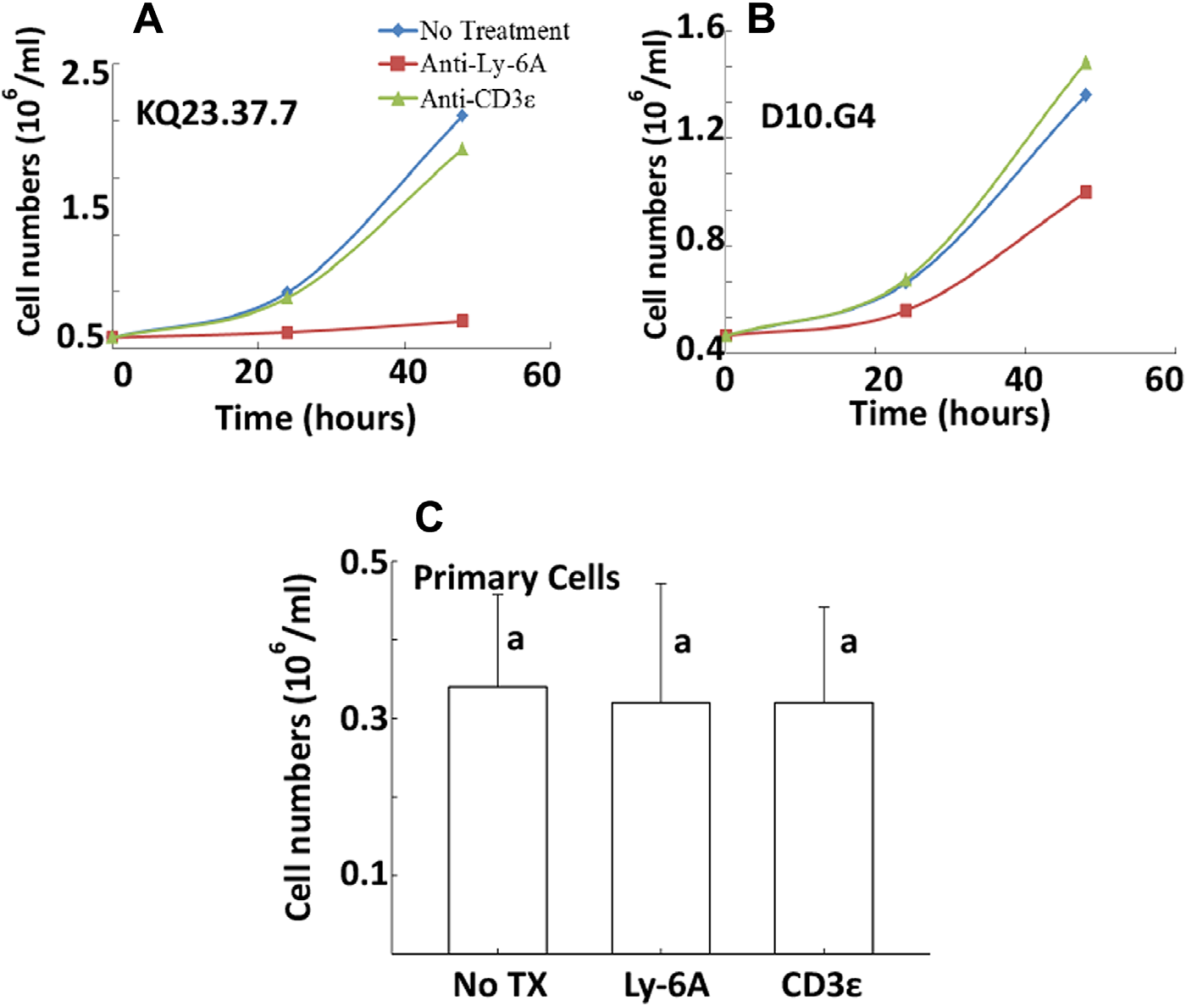
Viability of CD4^+^ T‐cell lines and primary lymph node cells after treatment with anti‐Ly‐6A/Sca‐1 antibody. KQ23.37.7 (A), D10.G4 (B), and primary lymph node (C) cells were treated with anti‐Ly‐6A/Sca‐1 (8G12) (Red Square), anti‐CD3ϵ (145‐2C11) (Green Triangle) mAbs or left untreated (Blue Diamond) for varied time (A and B) or 24 h (C) and live cells were enumerated as described in materials and methods section. A representative graph of total three independent trials is shown (A and B). Average count of live lymph node cells (*N* = 3) is shown (C). Kruskal–Wallis ANOVA was used to analyze the data in (C), and there was no significant difference found between antibody‐treated and control groups: Chi squared = 0.1927, *p*‐value = 0.9801; Chi squared = 0.5178, *p*‐value = 0.7719 are reported for anti‐Ly‐6A/Sca‐1 and anti‐CD3ϵ responses, respectively. The same letter denotes no statistical difference between the untreated and treated groups.

The growth inhibitory effects of anti‐Ly‐6A/Sca‐1 was tested on other CD4^+^ T cell lines and normal lymph node T cells. Similar effects were observed in KQ23.23.7 and D10.G4, two other independently‐derived CD4^+^ T cell lines (Fig. 3A and B). A representative experiment (*n* = 3) is shown (Fig. 3A and B). However the extent of growth inhibition differed between the two cell lines. While KQ23.37.7 showed similar growth inhibition as the YH16.33 cell line, the D10.G4 with about 40–50% lower expression of Ly‐6A/Sca‐1 (data not shown) showed reduced growth inhibition (Fig. 3B). In contrast, anti‐Ly‐6A/Sca‐1 antibody did not show growth inhibitory response on primary lymph node T cells (Fig. 3C). Taken together, our results suggest that growth inhibitory effects of engaging Ly‐6A/Sca‐1 protein are specific to transformed CD4^+^ T cells and not on primary T cells from the lymph node.

### Engaging Ly‐6A/Sca‐1 induces expression of p27^kip^ without affecting p53 expression

**Figure 4:**
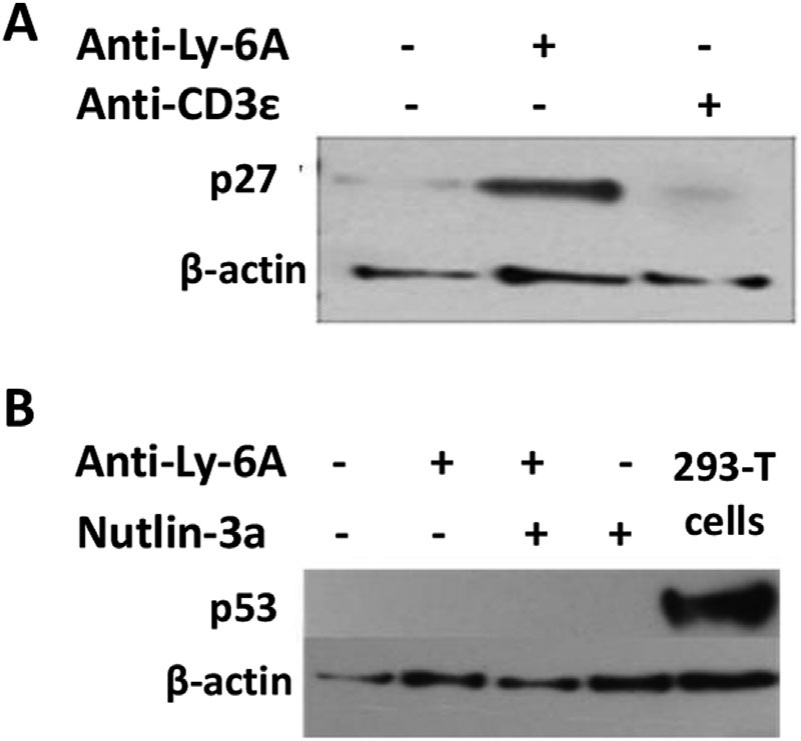
Expression of cell cycle proteins in YH16.33 cells in response to anti‐Ly‐6A/Sca‐1 mAb. Lysates of YH16.33 cells incubated with anti‐Ly‐6A/Sca‐1 mAb generated at 24 h time point were examined for the expression of p27^kip^ (A) and p53 (B) proteins by Western blots. Additionally, lysates from YH16.33 cells treated with Nutlin‐3a, at 10 µg/ml final concentration, were examined for p53 protein (B). Lysates from 293T cell line were used as positive control (B). Images are representative of three independent trials.

Growth inhibition in a variety of cell types involves up‐regulation of p27^kip^, a cell cycle inhibitory protein 30. We next sought to examine p27^kip^ expression in YH16.33 cells after their Ly‐6A/Sca‐1 protein was engaged. Figure 4A shows that p27^kip^ protein was present at about equal level in YH16.33 cells treated with anti‐CD3ϵ or left untreated. Expression of p27^kip^ was increased by more than 10‐fold when YH16 cells were treated with anti‐Ly‐6A/Sca‐1 antibody. In these experiments the expression of p53 was not observed in any of the above treatments (Fig. 4B). In addition, we tested expression of p53 in YH16.33 cells treated with anti‐Ly‐6A/Sca‐1 and Nutlin 3a, as the latter is known to inhibit degradation of p53 by blocking the binding of ubiquitin ligase mouse double minute 2 (MDM2) to p53 protein 31. We were unable to observe p53 protein in lysates of YH16.33 cells treated with a combination of anti‐Ly‐6A/Sca‐1 and Nutlin 3a by Western blots (Fig. 4B). The absence of detection was not due to the inactivity of the anti‐p53 antibody used in our experiments as we were able to detect p53 in lysates from 293T cells. These results indicate that cross‐linking of Ly‐6A/Sca‐1 protein results in up‐regulation of a cell cycle inhibitory protein, p27^kip^. Additionally, in its steady state‐level, p53 protein was undetectable in YH16.33 cells.

### Ly‐6A/Sca‐1 mAb activates caspase‐3

**Figure 5:**
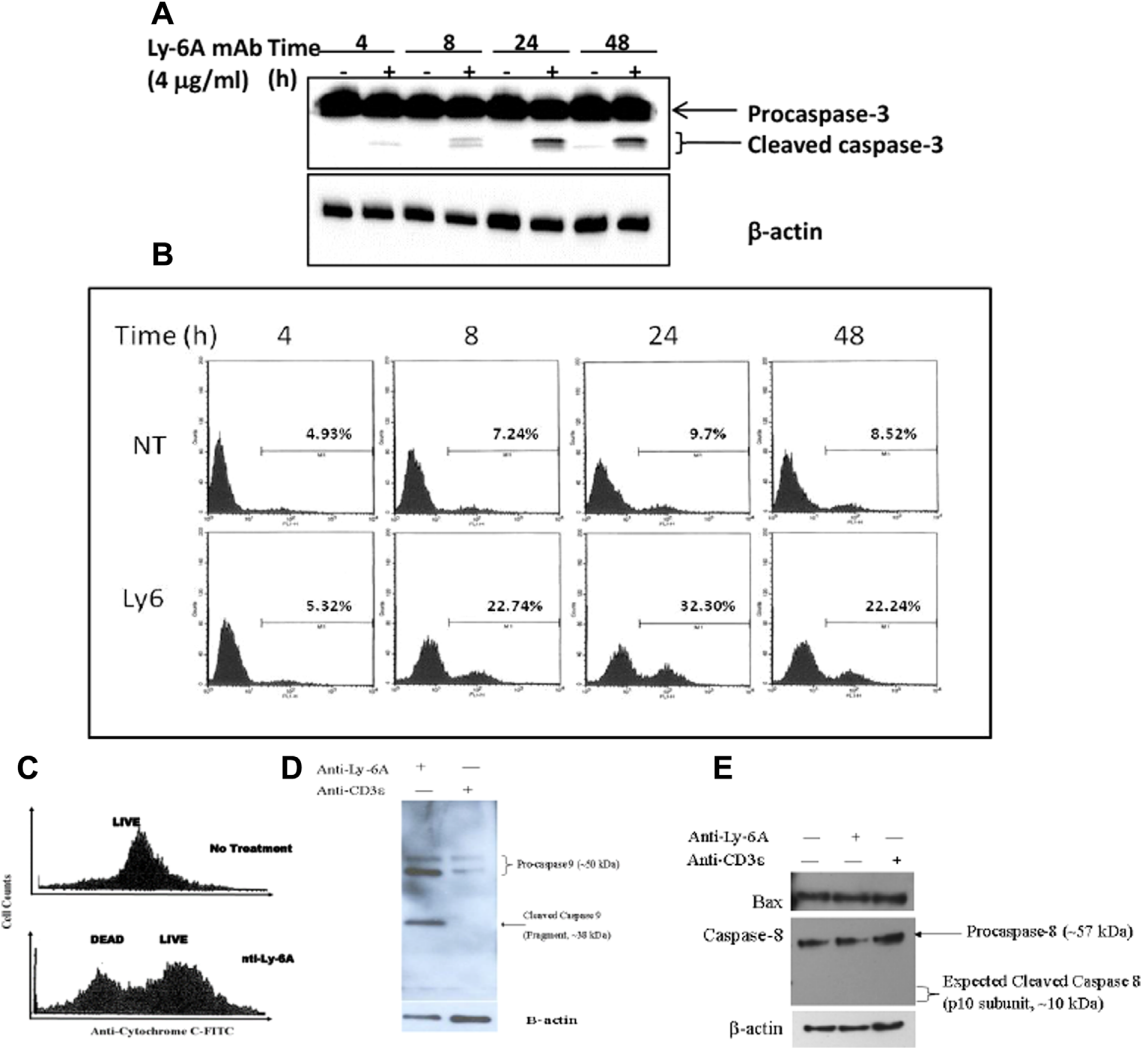
Activation of Caspase 3 in YH16.33 cells after Ly‐6A/Sca‐1 mAb treatment. (A and B) Cells were incubated with media alone (NT) or with anti‐Ly‐6A/Sca‐1 monoclonal antibody (Ly‐6) for 4, 8, 24, or 48 h and the presence of cleaved caspase 3 was detected in the cell lysates by Western blotting using anti‐caspase 3 antibody (A) and flow cytometry (B). Flow cytometeric analysis was carried out after staining with FITC‐labeled DEVD‐FMK which binds to activated caspase‐3. Cells positively stained for the marker were then analyzed. Percent gated show cells undergoing cell death with active intracellular caspase 3. (C) Release of cytochrome C from the mitochondria into the cytoplasm was detected by flow cytometer in anti‐Ly‐6A/Sca‐1 antibody treated YH16.33 cells at 24 h time point. Under same experimental conditions cleaved pro‐caspase 9 (D), caspase 8 (E), and Bax (E) was detected by western blots. Data shown are representative of three independent trials.

To further confirm that pro‐apoptotic signals are induced by engaging Ly‐6A/Sca‐1 proteins, we examined the activation of caspase‐3 using both Western blot and flow cytometric analyses. The activation of a family of cysteine‐protease caspase(s) is one of the crucial events in apoptosis. One particular caspase, caspase‐3, is a vital executioner of apoptosis as it is responsible for the proteolytic cleavage of endogenous proteins that are vital for signal transduction and structural maintenance of the cell 24. Caspase‐3 is activated by proteolytic processing of its inactive zymogen form into its p17 and p12 catalytically active fragments 25. As seen in Figure 5A, Western blot analysis detected the cleaved (active) fragments of caspase‐3 as early as 4 h after Ly‐6A/Sca‐1 mAb treatment. The active caspase‐3 proteins fragments detected increased in the Ly‐6A/Sca‐1 mAb treatment group over the time course tested. In contrast, active caspase‐3 fragments from the control group were not seen in early time points (data not shown). To corroborate the Western blot analysis, we also performed flow cytometry on YH16.33 cells stained with a FITC‐labeled DEVD‐FMK (FITC‐DEVD‐FMK) marker that binds irreversibly to active caspase‐3. After 8 h of Ly‐6A/Sca‐1 mAb treatment, the percentage of YH16.33 cells that positively stained for the FITC‐DEVD‐FMK marker was 22.74% (Fig. 5B). While only 7.24% positively stained cells were seen in the control group at the same time period. This disparity in the percentage of FITC‐DEVD‐FMK^+^ cells between the control and Ly‐6A/Sca‐1 mAb group was also observed in the 24 and 48 h treatment time periods (9.7–32.3%, and 8.52–22.24%, respectively). As a result, from the data in Figure 5A and B, we conclude that the pro‐apoptotic signal that is generated by cross linking Ly‐6A/Sca‐1 proteins can rapidly activate caspase‐3.

We next examined the role of caspase 8 in apoptotic cell death induced by anti‐Ly‐6A/Sca‐1. Caspase 8 is an initiator caspase that typically is recruited by the death receptors to the membrane to trigger cell‐extrinsic cell death, a pathway distinct from cell‐intrinsic death pathway triggered by the release of cytochrome C from the mitochondria. We did not detect, in our Western blots, the active fragment of caspase 8 with a specific rabbit monoclonal antibody directed against caspase 8 in YH16.33 cells exposed to anti‐Ly‐6A/Sca‐1 antibody (Fig. 5E), nor did we observe changes in the levels of Bax, a pro‐apoptotic protein, when compared to the controls (Fig. 5E). In contrast, the Ly‐6A/Sca‐1 engagement triggers the release of cytochrome C as assessed by intracellular staining with anti‐cytochrome C antibody followed by flow cytometeric analysis (Fig. 5C) and generates active caspase 9 (Fig. 5D). These data indicated that apoptotic cell death through cell intrinsic pathway is triggered in the YH16.33 T cell line when Ly‐6A/Sca‐1 is engaged.

## Discussion

In the present study, we report that engaging Ly‐6A/Sca‐1, a GPI‐anchored protein, on clonal CD4^+^ T cells triggers cytokine response, growth inhibition, and apoptosis through distinct mechanisms. While production of IL‐2 by immortalized CD4^+^ T cells in response to engaging Ly‐6A/Sca‐1 protein was dependent on the lipid raft‐based membrane order, the apoptotic cell death was independent of lipid raft integrity. In addition, cells undergoing apoptotic cell death generated signatures associated with cell‐intrinsic apoptosis.

Cell death triggered after engaging Ly‐6A/Sca‐1 protein with a monoclonal antibody showed signatures of apoptosis. This inference is based on assessing the asymmetry of plasma membrane of anti‐Ly‐6A/Sca‐1 treated cells by staining with Annexin V‐FITC followed by flow cytometeric analysis (Fig. 2) and by detecting intracellular activated caspase‐3 (Fig. 5A and B). It is generally accepted that the apoptotic cascade proceeds through two distinct pathways [reviewed in 32]. The “extrinsic pathway” of apoptosis is initiated when the death receptors on the plasma membrane are engaged by their ligands in either the soluble or cell surface bound forms 32, resulting in the formation of death‐inducing signaling complex (DISC) 24. Activation induced cell death in T cells and exposure to chemotherapeutic compounds are known to induce apoptotic cell death in T cell lines by recruiting death receptors and initiator caspases to DISC 24, 33, 34. Disruption of lipid rafts interferes with apoptosis induced through DISC where the Fas receptor and a number of initiation caspases (Caspase‐8, 9, and 10) play a central role 35, 36. In contrast, anti‐Ly‐6A/Sca‐1 triggered apoptosis was not inhibited when integrity of lipid rafts was disrupted with 7‐KC (Fig. 2), therefore suggesting a different mechanism underlying cell death initiated by engaging Ly‐6A/Sca‐1 protein. Consistent with this data are our Western blot experiments, where we did not detect active caspase 8, an initiator caspase recruited by the death receptor (Fig. 5E). Together these observations suggest that the pro‐apoptotic Ly‐6A/Sca‐1 signal does not utilize initiator caspases associated with extrinsic apoptotic cell death pathway. Detection of intracellular cytochrome C (Fig. 5C) suggests that by engaging Ly‐6A/Sca‐1, the mitochondrial membrane loses permeability resulting in release of cytochrome C that in turn triggers the activation of caspase 3 and 9 (Fig. 5A, B, and D), the executioners of apoptosis. These data support the idea that Ly‐6A/Sca‐1 triggers an “intrinsic apoptotic pathway,” however further experiments are required to investigate how a tail‐less Ly‐6A/Sca‐1 protein on the outer leaflet of the membrane connects to the mitochondria present in the cell interior. Either direct signaling by Ly‐6A/Sca‐1 protein into mitochondria or an indirect mechanism, where signaling through Ly‐6A/Sca‐1 results in production of a death factor that in turn initiates intrinsic cell death pathway are two possibilities for investigation in the future.

Direct engagement of Ly‐6A/Sca‐1 proteins with cross‐linking antibodies activated immortalized CD4^+^ T cell lines as assessed by their cytokine secretion 9, 10. How a tail‐less GPI‐anchored, Ly‐6A/Sca‐1 protein communicated with the cell interior has remained unclear. Published reports suggest that Ly‐6A/Sca‐1 requires a transmembrane protein complex for its signaling 20, 37. Surface expression of a transmembrane protein complex, the TCRαβ/CD3 20 and cytoplasmic domain of one of its component, TCR‐ζ, [reviewed in 37] are critical for responses through GPI‐anchored proteins (Ly‐6A/Sca‐1 and Thy‐1) in CD4^+^ T cells. However, physical associations between the antigen receptor and its components with Ly‐6A/Sca‐1 proteins have been hard to establish over decades of experimentation (unpublished results). GPI‐anchored proteins, such as Ly‐6A/Sca‐1 are localized to lipid rafts in a number of cell types, including T lymphocytes 38, 39, 40. Co‐opting membrane domains like lipid rafts where signaling receptors (like TCR/CD3) and other signaling proteins assemble and can potentially interact with GPI‐anchored proteins for its signaling remains to be fully explored. This idea is consistent with the reports that lipid rafts can coalesce to form signaling platforms to organize and recruit proteins that are involved in various cell signaling conduits, such as the apoptotic pathway 35 as well as cell activation 41. Additionally, a co‐immunoprecipitation study has indicated that GPI‐anchored proteins (like Ly‐6A/Sca‐1) associate with p56^lck^ and p59^fyn^, the two key protein tyrosine kinases located in lipid rafts 42. We have proposed that instead of their direct association, the co‐immunoprecipitation experiments perhaps suggest their indirect association through their localization in lipid rafts 27. Our experiments reported here show that YH16.33 T cells with compromised lipid raft‐based membrane order produce reduced levels of IL‐2 in response to anti‐Ly‐6A/Sca‐1 mAbs (Fig. 1). Further experimentation is required to identify a transmembrane protein(s), (TCRαβ/CD3 or others) housed in lipid rafts that possibly can connect Ly‐6A/Sca‐1 present on the outer leaflet of the membrane to the signaling kinases (e.g., p56^lck^ and p59^fyn^) tethered to the inner leaflet, and therefore aid in, a better understanding of the signaling pathway triggered through the tail‐less, GPI‐anchored proteins.

The growth inhibitory effect of anti‐Ly‐6A/Sca‐1 antibody has been described previously 37, 43. These inhibitory effects are not due to down‐regulation of the protein expression. Activated CD4^+^ T cells and transformed CD4^+^ T cells express high level of Ly‐6A protein 2. While the growth inhibitory effect of anti‐Ly‐6A/Sca‐1 are known, but cell cycle proteins involved in this process were not reported. We speculated that the p53 protein was involved in the growth inhibition and cell death observed in YH16.33 cells after engaging Ly‐6A/Sca‐1 protein on its plasma membrane. In transformed cells, p53 is often mutated, ubiquinated by MDM2 for degradation, thus preventing it from performing its normal function (cell cycle arrest) resulting in uncontrollable cell growth 44, 45. The observation of growth inhibition and apoptotic cell death in YH16.33 cells after engagement of Ly‐6A/Sca‐1 proteins prompted us to examine the expression of p53 in YH16.33 cells in the presence and absence of anti‐Ly‐6A/Sca‐1 mAb. In some of these experiments Nutlin‐3a, an inhibitor of MDM2 E3 ligase was included, given its role in inhibiting the interaction between p53 and MDM2 31. We did not detect p53 protein in YH16.33 cells at its steady‐state level either in the absence of or presence of anti‐Ly‐6A mAb (Fig. 4B). p53 protein was undetectable in YH16.33 cells even after pharmacological treatement of YH16.33 cells with Nutlin 3a at 10 µg/ml concentration (Fig. 4B). Absence of detectable p53 in YH16.33 lysates strongly suggest that Ly‐6A/Sca‐1 specific growth inhibition observed in YH16.33 cells occur in the absence of p53 tumor suppressor gene. However, we observed an up‐regulated expression of p27^kip^, a cell cycle inhibitor protein instead (Fig. 4A). P27^kip^ regulates activity of cyclin‐dependent kinase in response to anti‐mitotic signaling 46, 47, 48. High expression of p27^kip^ is associated with differentiation phenotype of a variety of cell types and low expression with embryonic stem cell phenotype in human embryonic stem cells by regulating expression of *Brachyury* and *Twist* 49. Further experimentation will be required to identify downstream targets of p27^kip^ in T cell lines and link between growth inhibition and cell death by apoptosis, if any. The growth inhibitory response observed after engaging Ly‐6A/Sca‐1 protein by cross‐linking with a monoclonal antibody appears to mirror the growth inhibitory role of this protein on immune and non‐immune cells. CD4^+^ T cells overexpressing Ly‐6A/Sca‐1 protein show reduced proliferation in response to a specific antigen 13 and CD4^+^ T cells lacking the expression of Ly‐6A/Sca‐1 protein show modest hyper‐proliferation 12. These findings are consistent with a role of Ly‐6A/Sca‐1 protein in regulating cell signaling and proliferation through the antigen receptor in primary CD4^+^ T cells. Ly‐6A/Sca‐1 induced expression of a p27^kip^ suggests a role of this cell cycle inhibitory protein in the growth inhibition and provides rationale to investigate this further.

While expression of Ly‐6A/Sca‐1 on primary T cells influences signaling through the antigen receptor, direct engagement of Ly‐6A/Sca‐1 alone does not generate cell death and growth inhibition responses (Unpublished data and Fig. 4). In contrast, directly engaging Ly‐6A/Sca‐1 on transformed CD4^+^ T cell lines show measurable cellular responses, thus suggesting that Ly‐6A/Sca‐1 signaling pathways are likely to be wired differently between primary and immortalized CD4^+^ T cells. Ly‐6A/Sca‐1 protein is highly expressed on transformed cells and its expression co‐relates to the tumorigenic potential of these cells 50, 51, 52. Prostate stem cell antigen (PSCA), a member of the Ly‐6 family is overexpressed in prostate cancer cells 50. In addition, the Ly‐6A/Sca‐1 protein appears to regulate tumorigenesis 53, 54. The high expression level and the growth inhibitory/cell death inducing properties of Ly‐6A/Sca‐1 in transformed cells provide an opportunity to target Ly‐6A/Sca‐1 for cancer therapy.

## Conflicts of Interest

The authors declare no commercial or financial conflicts of interest.

## Supporting information


**Figure S1**. 7‐KC effects membrane order in YH16.33 CD4^+^ T cell line. YH16.33 were incubated with di‐4 ANEPPDHQ dye. T cells treated with 58 μM (C), 29 μM (D), 14.5 μM (E), mβCD‐vehicle control (F), or were not exposed to 7‐KC (B). di‐4 ANEPPDHQ stained cells were examined by flowcytometer by exciting the dye at 488 nm and assessing emission at 570 nm (FL2 channel) and 630 nm (FL3 channel). YH16.33 cells left unstained (panel A) were used as negative control to set‐up the FACS. Generalized polarization (GP) values for 7‐KC treated and the control mβCD treated cells was computed based on the mean fluorescence intensity of emission at 570 and 630 nm. 7‐KC treated or control mβCD treated control cells were plotted (lower panel). This graphic representation of three independent experiments is shown (Lower panel), Statistical significance between untreated, 7‐KC test and mβCD control groups was computed by two way ANOVA using JMP program. Different lower case alphabet designations (a–c) above each treatment group (no treatment, 58 μM 7‐KC, 29 μM 14.5 μM, mβCD) indicates statistically significant difference (*p *< 0.05). Similar lower case alphabet designation indicates lack of statistical significance (*p* > 0.05).

**Figure S2**. Expression of CD4^+^ T cell molecules on the surface of either 7‐KC treated or untreated cells by Flow cytometry is shown. YH16.33 cells treated with 7‐KC for 15 min at RT or left untreated were stained with either PE or FITC conjugated anti‐Ly‐6A/Sca‐1, anti‐CD3ϵ, anti‐TCRϵβ, and anti‐LFA‐1 monoclonal antibodies and analyzed by flow cytometer. Histograms shown represents the two experiments.

**Figure S3**. Growth inhibition in anti‐Ly‐6A/Sca‐1 antibody treated cells. YH16.33 or control MVB2 cells were treated with either anti‐Ly‐6A/Sca‐1 (8G12) or anti‐CD3ϵ (145‐2C11) for 48 hours and cell cultures were assessed for growth and survival by MTS assay. Absorbance at 490 nm was measured in triplicate samples. The data represent the mean ± S.D. of 7 independent experiments. Groups denoted with different letters are statistically different (*p* < 0.05).
