# Supplementary figures and images for: Engaging Ly‐6A/Sca‐1 triggers lipid raft‐dependent and ‐independent responses in CD4+ T‐cell lines
Source: Immun Inflamm Dis. 2017 Jun 28;5(4):448–60. doi: 10.1002/iid3.182 (PMC5691314; doi:10.1002/iid3.182)

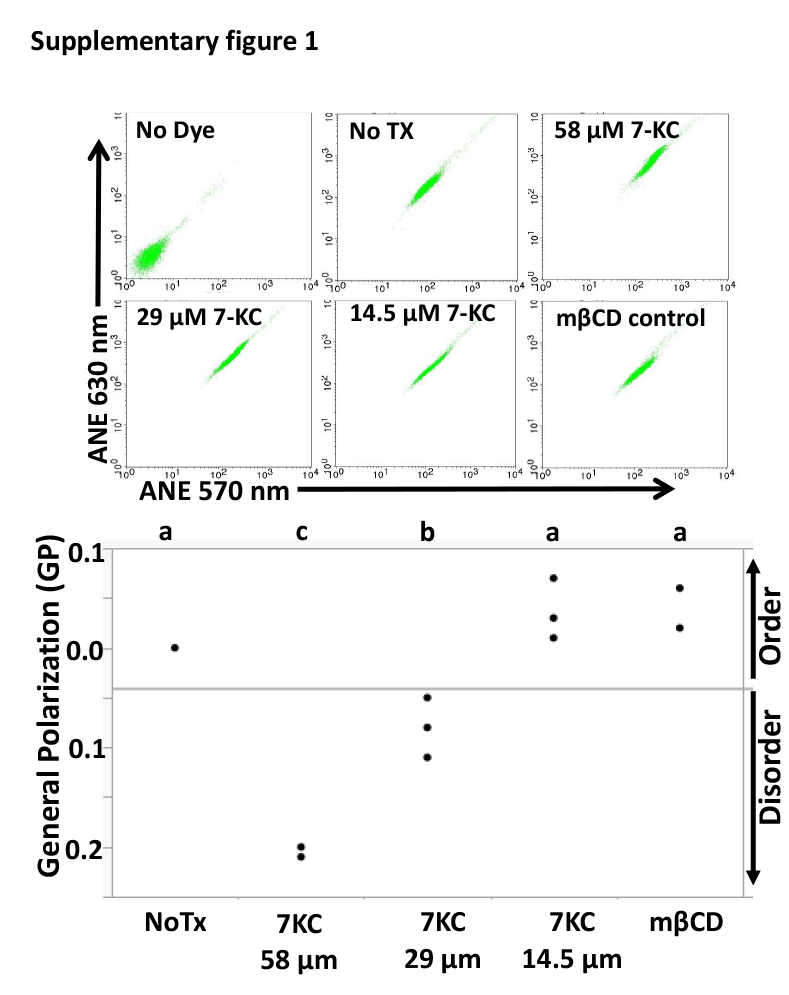

Supplement: Supplementary file 1 — Figure S1. 7‐KC effects membrane order in YH16.33 CD4+ T cell line. YH16.33 were incubated with di‐4 ANEPPDHQ dye. T cells treated with 58 μM (C), 29 μM (D), 14.5 μM (E), mβCD‐vehicle control (F), or were not exposed to 7‐KC (B). di‐4 ANEPPDHQ stained cells were examined by flowcytometer by exciting the dye at 488 nm and assessing emission at 570 nm (FL2 channel) and 630 nm (FL3 channel). YH16.33 cells left unstained (panel A) were used as negative control to set‐up the FACS. Generalized polarization (GP) values for 7‐KC treated and the control mβCD treated cells was computed based on the mean fluorescence intensity of emission at 570 and 630 nm. 7‐KC treated or control mβCD treated control cells were plotted (lower panel). This graphic representation of three independent experiments is shown (Lower panel), Statistical significance between untreated, 7‐KC test and mβCD control groups was computed by two way ANOVA using JMP program. Different lower case alphabet designations (a–c) above each treatment group (no treatment, 58 μM 7‐KC, 29 μM 14.5 μM, mβCD) indicates statistically significant difference (p < 0.05). Similar lower case alphabet designation indicates lack of statistical significance (p > 0.05). [file IID3-5-448-s001.tif]

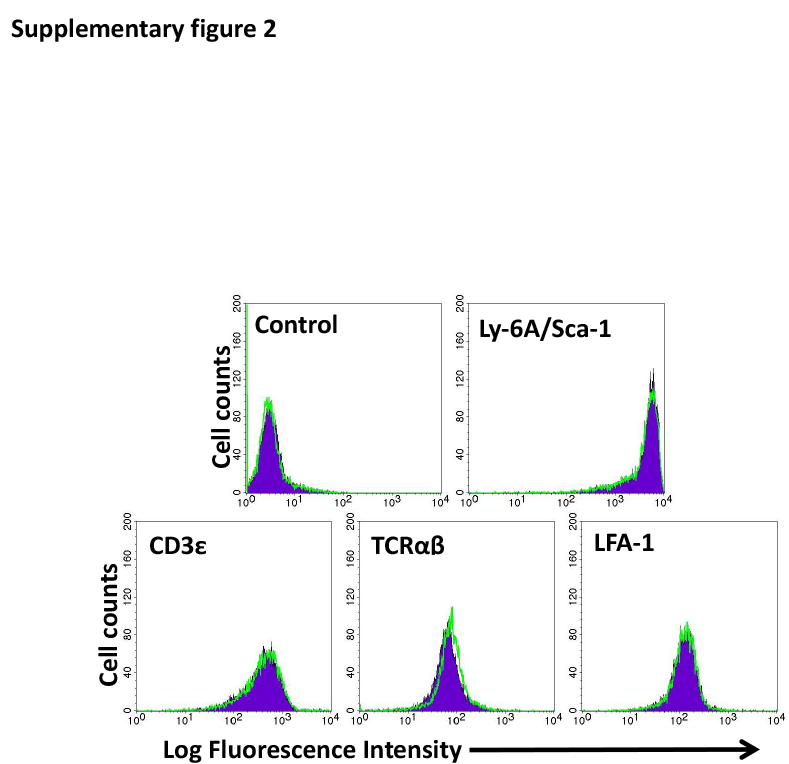

Supplement: Supplementary file 2 — Figure S2. Expression of CD4+ T cell molecules on the surface of either 7‐KC treated or untreated cells by Flow cytometry is shown. YH16.33 cells treated with 7‐KC for 15 min at RT or left untreated were stained with either PE or FITC conjugated anti‐Ly‐6A/Sca‐1, anti‐CD3ϵ, anti‐TCRϵβ, and anti‐LFA‐1 monoclonal antibodies and analyzed by flow cytometer. Histograms shown represents the two experiments. [file IID3-5-448-s002.tif]

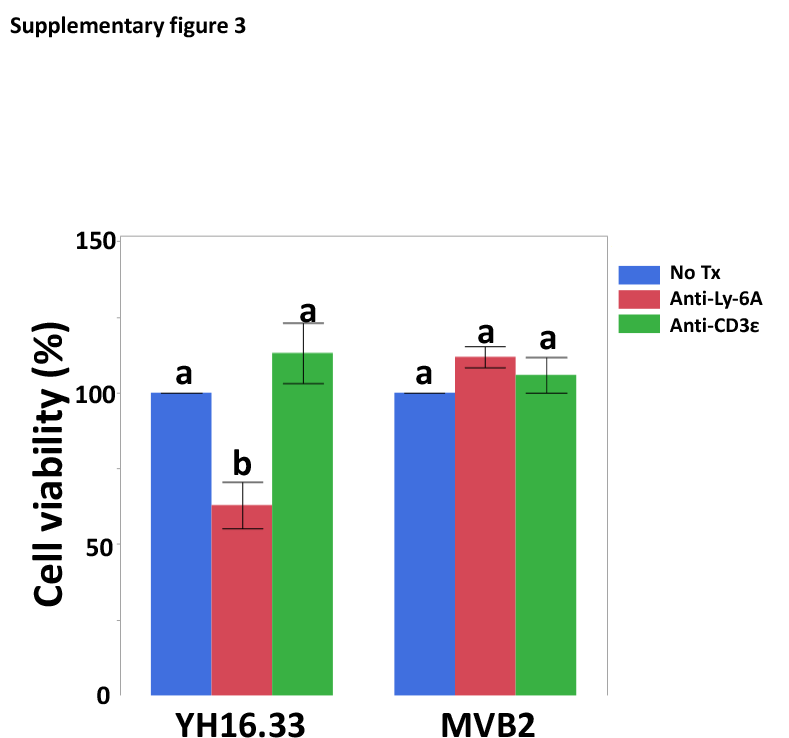

Supplement: Supplementary file 3 — Figure S3. Growth inhibition in anti‐Ly‐6A/Sca‐1 antibody treated cells. YH16.33 or control MVB2 cells were treated with either anti‐Ly‐6A/Sca‐1 (8G12) or anti‐CD3ϵ (145‐2C11) for 48 hours and cell cultures were assessed for growth and survival by MTS assay. Absorbance at 490 nm was measured in triplicate samples. The data represent the mean ± S.D. of 7 independent experiments. Groups denoted with different letters are statistically different (p < 0.05). [file IID3-5-448-s003.tif]
